# A Physics-Informed Generative Car-Following Model for Connected Autonomous Vehicles

**DOI:** 10.3390/e25071050

**Published:** 2023-07-12

**Authors:** Lijing Ma, Shiru Qu, Lijun Song, Zhiteng Zhang, Jie Ren

**Affiliations:** School of Automation, Northwestern Polytechnical University, Xi’an 710072, China; qushiru@nwpu.edu.cn (S.Q.);

**Keywords:** car-following modeling, hybrid models, generative model, deep learning, connected and autonomous vehicles, mixed traffic flow

## Abstract

This paper proposes a novel hybrid car-following model: the physics-informed conditional generative adversarial network (PICGAN), designed to enhance multi-step car-following modeling in mixed traffic flow scenarios. This hybrid model leverages the strengths of both physics-based and deep-learning-based models. By taking advantage of the inherent structure of GAN, the PICGAN eliminates the need for an explicit weighting parameter typically used in the combination of traditional physics-based and data-driven models. The effectiveness of the proposed model is substantiated through case studies using the NGSIM I-80 dataset. These studies demonstrate the model’s superior trajectory reproduction, suggesting its potential as a strong contender to replace conventional models in trajectory prediction tasks. Furthermore, the deployment of PICGAN significantly enhances the stability and efficiency in mixed traffic flow environments. Given its reliable and stable results, the PICGAN framework contributes substantially to the development of efficient longitudinal control strategies for connected autonomous vehicles (CAVs) in real-world mixed traffic conditions.

## 1. Introduction

The field of car-following behavior modeling has garnered significant attention in the transportation community over several decades. As advancements in technology, such as machine learning, sensors, and communication systems, facilitate the development of connected and autonomous vehicles (CAVs), understanding and predicting human driving behavior becomes increasingly important. This is particularly true during the transitional period when CAVs coexist with human-driven vehicles (HVs). In this context, the development of precise car-following models is crucial to advancing the performance of autonomous driving systems, augmenting traffic safety, and promoting efficiency.

Conventional car-following models can be classified into two main categories: physics-based models and data-driven models. Physics-based models attempt to represent car-following behavior using established physical laws or empirical rules, offering interpretable and stable predictions. However, their reliance on simplified mathematical functions and a restricted number of parameters may limit their generalization across various driving scenarios. In contrast, data-driven models are capable of extracting underlying patterns from data without requiring prior knowledge or assumptions. While these models can effectively capture complex driving behaviors, they may occasionally produce physically inconsistent predictions. Therefore, hybrid car-following models have gained considerable attention in recent years. These hybrid models combine the strengths of both physics-based and data-driven approaches, incorporating the robustness of physical laws and the adaptability of machine learning methods to enhance predictive performance. This hybrid approach aims to achieve a balance between interpretability, generalizability, and accuracy in predicting car-following behavior by integrating elements from conventional models, especially in the context of CAVs.

Physics-informed deep learning (PIDL) is a prominent research area, with numerous studies exploring its potential applications and benefits [1,2,3]. In PIDL, physical knowledge is typically incorporated into deep neural networks through governing equations, physical constraints, or regularity terms in loss functions [4]. In terms of driving behavior modeling, groundbreaking work by Mo et al. [5] and subsequent studies [6,7,8,9] have established the PIDL paradigm as a promising avenue, demonstrating the advantages of leveraging existing physics-based car-following models within deep neural networks to enhance the training and prediction of car-following behavior.

While the studies mentioned above have made significant strides in combining physics-based models with deep learning architectures, some potential research gaps can be identified:

- In the design of hybrid models, an explicit weighting parameter is often employed to balance the contributions of physics-based and data-driven models or represent the weight for loss terms in the loss function. Although this approach enables the optimization of the hybrid model’s performance by fine-tuning the balance between the two models, it can present challenges in parameter tuning and overfitting. This may lead to increased computational costs and poor generalization to unseen data. Furthermore, this approach may reduce the model’s adaptability to new situations due to its inability to capture inherent dynamics between physics-based and data-driven models.
- When developing hybrid car-following models for CAVs, the incorporated physics-based models are often general models that simulate human driving behavior, such as the intelligent driver model (IDM) and the optimal velocity model (OVM). However, since these models are not specifically tailored for CAVs, their integration may impact the performance of the hybrid model.
- While recent studies have demonstrated the potential of hybrid car-following models to improve the performance of CAVs, there remains a need for the extensive evaluation and validation of these models in mixed traffic flow. This would involve high-fidelity simulations that consider the complex interactions between CAVs and HVs, as well as diverse driving scenarios and environments.

To address the above challenges, we propose a novel hybrid car-following model called the Physics-Informed Conditional Generative Adversarial Network (PICGAN). It integrates a physics-based model into the training process of the Conditional Generative Adversarial Network (CGAN) structure. In essence, it utilizes the physics-based model as an additional discriminator. This integration allows the model to leverage the strengths of the physics-based approach, namely, its interpretability and reliability under normal traffic conditions, as well as those of the data-driven approach, like its capability to capture complex nonlinear relationships and perform well under diverse traffic conditions. Our key contributions are as follows:

- The proposed PICGAN model eliminates the need for explicit weighting parameters, bringing together the benefits of both physics-based and deep-learning-based approaches. This unique combination enhances the adaptability and generalization capabilities of the car-following model.
- The hybrid framework incorporates a custom physics-based model designed specifically for CAVs, namely, the control model developed by the PATH laboratory based on actual vehicle implementation. This integration enhances the applicability and performance of the hybrid model in CAV scenarios.
- Numerical simulations are conducted to assess the performance of the proposed hybrid car-following model. Specifically, we employ a platoon simulation to verify the model’s stability, and we use a periodic boundary condition to gauge the model’s effectiveness in mixed traffic flow scenarios.

The subsequent sections of this paper are organized as follows: Section 2 provides an in-depth literature review. In Section 3, we present the architecture of the proposed physics-informed generative car-following model. Section 4 details the training of the proposed model using an empirical dataset, and it also presents the model’s performance based on prediction accuracy, platoon simulation, and mixed traffic flow simulation. In Section 5, our findings are discussed and concluded.

## 2. Literature Review

### 2.1. Conventional Car-Following Models

Car-following models, which describe the longitudinal interactions between adjacent vehicles, have been extensively studied for decades; the first model was proposed by Pipes nearly seventy years ago [10]. Physics-based car-following models can be broadly categorized into four groups: stimulus–response models [11,12,13,14,15,16], desired measures models [17,18,19,20], psycho-physical models [21,22], and Newell’s simplified models [23,24,25,26,27]. These models are built on various behavioral perspectives, such as stimulus–response, desired measures, perceptual thresholds, and parsimonious replication of vehicle trajectories, providing a foundation for understanding driver behaviors and capturing traffic oscillations.

Data-driven car-following models have gained traction in recent years as they leverage real-world traffic data and do not require mathematical formulas or calibration. These models employ various machine learning techniques to achieve more accurate and adaptable car-following behaviors. Nonparametric regression models use techniques like locally weighted regression [28,29] and k-nearest neighbor [30] to predict vehicle positions and reproduce traffic dynamics, offering a flexible and adaptive approach to car-following behavior modeling. Support vector regression (SVR) models [31] apply the principles of support vector machines (SVM) to regression problems. In car-following modeling, SVR considers inputs like space headway, the follower’s speed, and relative speed to output the follower’s speed, allowing the model to capture the asymmetric characteristics of car-following behavior. Reinforcement learning (RL) models use deep reinforcement learning to train agents to make car-following decisions. Flow, a computational framework [32], integrates traffic simulators and deep RL libraries to enable the development of controllers for autonomous vehicles in complex traffic scenarios. Other RL models [33,34,35] focus on learning personalized reward functions and driving strategies from historical driving data, leading to more human-like and adaptable car-following behaviors. Deep learning (DL) models can account for complex driving behavior patterns, resulting in more accurate traffic flow simulations. Studies have focused on supervised learning approaches, such as recurrent neural networks (RNN) [36], long short-term memory (LSTM) [37], encoder–decoder [38], and attention-based transformer models [39], to predict traffic oscillations and car-following trajectories. To address some of the deficiencies of supervised learning, Generative Adversarial Network (GAN)-based car-following models [40,41,42,43,44,45] have emerged, generating realistic and diverse car-following behaviors. Overall, these data-driven car-following models offer improved performance over physics-based models, showing significant potential for enhancing autonomous driving algorithms and traffic flow models.

### 2.2. Hybrid Car-Following Models

Hybrid car-following models have attracted significant attention in recent years due to their potential to combine the strengths of physics-based and data-driven models, enhancing the prediction of car-following behavior. It should be noted that physics-based models may be referred to by different names, such as kinematics-based, theoretically driven, model-based, and mathematical models. In this section, we review four kinds of studies that have developed hybrid car-following models, revealing common trends in this research area.

The fusion of machine learning techniques with classical car-following models is a common approach in the development of hybrid car-following models. Yang et al. [46] employed a combination forecast method to merge machine-learning-based and kinematics-based models, achieving superior performance by determining the optimal weight values between distinct car-following models. The results demonstrate that the combination car-following model outperforms individual models in terms of safety and robustness. Li et al. [47] put forth a fusion modeling approach that amalgamates data-driven and theoretically driven car-following models, constructing a combined LSTM–IDM car-following model using an adaptive Kalman filter. Wu and Work [48] focused on incorporating the structure of classical models into the neural networks by modifying architectures and activation functions. These approaches aim to achieve superior performance by optimally combining distinct car-following models, demonstrating the value of hybrid modeling in improving safety and robustness.

The integration of physics-based models into deep learning architectures has been a recurring theme in hybrid car-following models. Mo et al. [5] proposed a physics-informed deep learning (PIDL) paradigm for car-following models that combines the strengths of both physics-based models and deep learning models. The combination process involves encoding physics-based models into deep neural networks to create a family of neural network-based car-following models that are informed by physics-based models. This approach aims to improve the accuracy and data efficiency of car-following behavior prediction by integrating fundamental traffic flow theories from physics-based models into deep learning architectures. In accordance with this paradigm, Xu et al. [6] integrated an improved IDM into PIDL and proposed a complementary fusion model for car-following prediction. To address uncertainty quantification problems in car-following behaviors, Mo and Di [7] encoded the stochastic physics into the PIDL structure. This model adds a physics discrepancy term to the loss function of GAN, to learn the underlying physics of car-following behaviors and generate realistic samples of driver trajectories under different uncertainty scenarios. Wang and Feng [8] integrated the IDM with a sequence-to-sequence recurrent autoencoder, enabling the simultaneous prediction of the multi-step trajectory. This integration is facilitated by the design of a hybrid loss function that includes the calibrated car-following model during the autoencoder training process. Naing et al. [9] proposed a jointly trained approach, allowing for a more powerful hybrid car-following model that can capture both short-term dynamics and long-term trends in traffic flow. Using the IDM as a constraint to guide the learning process, the LSTM is trained to learn underlying patterns in the data while ensuring that these patterns are consistent with real-world driving behaviors. These studies demonstrate the potential of combining the interpretability and domain knowledge of physics-based models with the flexibility and learning capabilities of deep learning models.

Reinforcement-learning-based car-following models have been combined with traditional car-following strategies to develop hybrid car-following approaches. Yan et al. [49] proposed a hybrid car-following strategy combining deep deterministic policy gradient (DDPG) and cooperative adaptive cruise control (CACC), addressing performance limitations by selecting optimal actions in real-time under the Markov decision process (MDP) framework and using a switching rule for smooth transitions. Yavas et al. [50] proposed a hybrid car-following policy that combines classical car-following policy with model-based reinforcement learning (MBRL) policy. The classical policy generates a reference acceleration, while the MBRL policy predicts future states of other vehicles and their trajectories, modifying the reference acceleration for more accurate and predictive control.

Some studies have also focused on developing hybrid car-following models by integrating probabilistic approaches with established car-following models. Soldevila et al. [51] integrated a parametric and a nonparametric mathematical formulation to predict individual drivers’ acceleration given a set of variables. The model uses Gaussian process regression (GPR) to make predictions when there is a correlation between new input and the training dataset. Zhang et al. [52] proposed a hybrid car-following model that integrates the IDM with time-varying parameters and a neural-process-based car-following model. By computing the aggressiveness index and mapping it to an intermediate variable of the neural process, the model can capture driving styles and generate realistic behavior.

In summary, the literature on hybrid car-following models reveals a growing interest in combining physics-based and data-driven models to enhance the prediction of car-following behavior. These studies highlight the value of hybrid modeling in advancing safety, robustness, and predictive accuracy within the car-following domain.

## 3. Methodology

### 3.1. Physics-Based Car-Following Models

Car-following models are designed to establish the mapping from the driving states, such as spacing headway, velocity differential, and velocity itself, to certain actions, typically acceleration and target velocity. Consider an input–output pair (X,Y), where *X* is an element of the state space S, and *Y* is an element of the action space A, i.e., X∈S and Y∈A. In the context of a physics-based car-following model, denoted as $f_{\lambda }$, the model is responsible for learning the mapping from the state space to the action space, represented as $f_{\lambda }:X\to Y$.

#### 3.1.1. Intelligent Driver Model

The intelligent driver model (IDM) was initially introduced in 2000 and has been successful in accurately reproducing phase transitions at road inhomogeneities [20]. This model is capable of capturing the car-following behaviors of human-driven vehicles, and its parameters possess distinct physical interpretations. The governing equation of IDM is expressed in Equations (1) and (2).

(1) $$a=\tilde{a}\left[1-{\left(\frac{v}{\tilde{v}}\right)}^{4}-{\left(\frac{s^{*}\left(v,\Delta v\right)}{\Delta x}\right)}^{2}\right]$$

(2) $$s^{*}\left(v,\Delta v\right)=s_{0}+\max\left(0,t_{0}v-\frac{v\Delta v}{2\sqrt{\tilde{a}\tilde{b}}}\right)$$

where *a* represents the acceleration of the subject vehicle. The relative speed, denoted by Δv, is calculated as the difference between the preceding vehicle’s speed (vp) and the subject vehicle’s speed (*v*), i.e., Δv=vp−v. The gap distance between adjacent vehicles is represented by Δx. $s^{*}$ is the desired gap distance function.

The model includes several parameters that characterize the driving behavior: the maximum acceleration ($\tilde{a}$), desired deceleration ($\tilde{b}$), desired speed ($\tilde{v}$), safe time gap ($t_{0}$), and minimum safe gap distance ($s_{0}$). These parameters need to be calibrated to accurately capture the car-following dynamics of human-driven vehicles.

#### 3.1.2. Cooperative Adaptive Cruise Control Model

The PATH laboratory has developed control models for adaptive cruise control (ACC) and cooperative adaptive cruise control (CACC) based on real vehicle implementation [53,54], demonstrating that ACC vehicle strings exhibit instability, while CACC strings enable smooth and stable following action. In this study, we concentrate on CAVs equipped with communication technology, which promotes the stability of mixed traffic flow. As the CACC model is derived from actual vehicle trajectories, it is employed to model CAVs. The control equations for the CACC model, including the gap error and the speed derivative, are presented in Equations (3) and (4).

(3) $$\left\{\begin{array}{c} e=\Delta x-s_{0}-t_{c}v\\ v=v_{p}+k_{p}e+k_{d}\dot{e} \end{array}\right.$$

(4) $$a=\frac{k_{p}\left(\Delta x-s_{0}\right)-k_{p}t_{c}v+k_{d}\Delta v}{k_{d}t_{c}+dt}$$

where *e* represents the gap error between the actual and desired gap. $t_{c}$ denotes the desired constant time gap. The speed of the subject vehicle at the previous control time is indicated by $v_{p}$. The control parameters $k_{p}$ and $k_{d}$ are utilized to adjust the time gap error. The updated time interval is represented by dt.

The values of $k_{p}$, $k_{d}$, and dt are determined through experimental tests [54], with $k_{p}=0.45$, $k_{d}=0.25$, and dt=0.01s. According to research in [55], the accepted values of $t_{c}$ vary, and the maximum acceptance rate is 57% when $t_{c}=0.6$.

### 3.2. Conditional GAN-Based (CGAN) Car-Following Model

Generative models focus on learning the underlying data distribution and generating new samples similar to the observed data. One powerful type of generative model is the Generative Adversarial Network (GAN), which consists of two neural networks, the generator (*G*) and the discriminator (*D*), which compete with each other in a min–max game. The generator’s goal is to create synthetic data samples that resemble the true data distribution, while the discriminator’s task is to distinguish between real samples from the true data distribution and fake samples generated by the generator. Conditional GAN (CGAN) [56] presents an advanced variation of the GAN framework, wherein the discrimination process is influenced by additional input variables. Integrating this additional information enables the generation of more refined and diverse outputs that align with specific conditions. Previous studies [44,45] have substantiated the practical utility of CGAN in the domain of car-following models.

**Figure 1:**
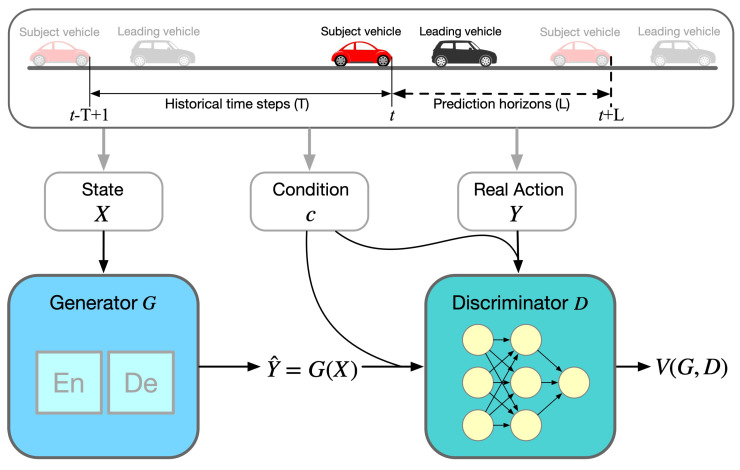
Architecture of CGAN-based car-following model.

The architecture of the CGAN for car-following modeling is illustrated in Figure 1. During the training process, the generator continually improves its ability to generate realistic actions, i.e., $\hat{Y}=G\left(X\right)$, while the discriminator enhances its ability to identify real action (*Y*) versus fake action ($\hat{Y}$), with the consideration of additional information (*c*). This adversarial process continues until an equilibrium is reached, where the generator produces samples that are indistinguishable from the real data, and the discriminator can no longer accurately differentiate between the two. The learning objective of the CGAN-based car-following model can be represented by Equations (5)–(7):

(5) $$D^{*}=\arg\max_{D}V\left(G,D\right)$$

(6) $$G^{*}=\arg\min_{G}\max_{D}V\left(G,D\right)$$

(7) $$V\left(G,D\right)=E_{\left(c,Y\right)∼P_{\mathrm{data}}}\left[\log D\left(c,Y\right)\right]+E_{c∼P_{\mathrm{data}},\hat{Y}∼P_{G}}[\log\left(1-D\left(c,\hat{Y}\right)\right]$$

where $D^{*}$ and $G^{*}$ represent the optimal discriminator and generator functions. V(G,D) represents the loss function that quantifies the difference between the actual data and the samples produced by the model. The symbol *E* stands for expectation. $P_{\mathrm{data}}$ signifies the true distribution of the data, while $P_{G}$ denotes the distribution obtained from the generator *G*. Two pairs are evaluated by the discriminator *D*, the pair (c,Y) comprising the condition and the real action, and the pair $\left(c,\hat{Y}\right)$ consisting of the condition and the generated action.

### 3.3. Physics-Informed CGAN-Based (PICGAN) Car-Following Model

#### 3.3.1. Architecture of PICGAN

**Figure 2:**
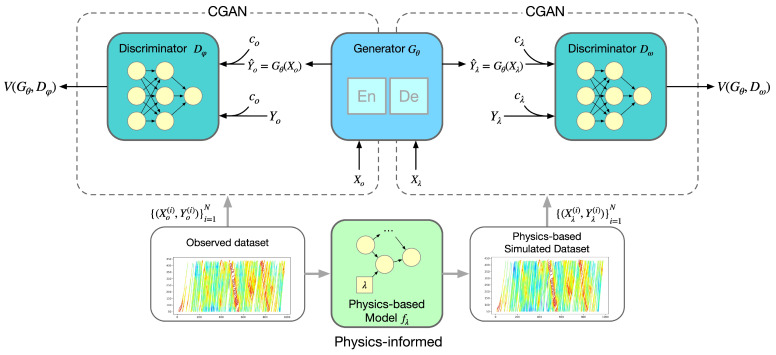
Architecture of PICGAN-based car-following model.

To leverage the advantages of both physics-based and data-driven models, we propose a physics-informed CGAN (PICGAN) architecture, as depicted in Figure 2. The PICGAN framework encompasses a generator and two discriminators, establishing two interlaced CGAN configurations that integrate a pre-trained physics-based model. The left CGAN structure is trained using observed data, while the right CGAN structure leverages data simulated from the physics-based model.

The generator ($G_{\theta }$) employs an encoder–decoder architecture, processing the input sequence ($X_{o}$/$X_{\lambda }$) to yield the output sequence (${\hat{Y}}_{o}$/${\hat{Y}}_{\lambda }$), as formulated in Equations (8) and (9). Concurrently, the condition sequence from the dataset ($c_{o}$/$c_{\lambda }$) serves as additional information for the discriminators ($D_{\varphi }$/$D_{\omega }$). The discriminator $D_{\varphi }$ learns to distinguish the output sequence from the real action of the observed sample and evaluates the consistency of the states in the observed sample, as represented by the loss function in Equation (10). Similarly, discriminator $D_{\omega }$ evaluates the output sequence against physics-based samples, with the loss function calculated in Equation (11).

(8) $${\hat{Y}}_{o}=G_{\theta }\left(X_{o}\right)$$

(9) $${\hat{Y}}_{\lambda }=G_{\theta }\left(X_{\lambda }\right)$$

(10) $$V\left(G_{\theta },D_{\varphi }\right)=E_{\left(c_{o},Y_{o}\right)∼P_{\mathrm{obs}}}\left[\log D_{\varphi }\left(c_{o},Y_{o}\right)\right]+E_{c_{o}∼P_{\mathrm{obs}},{\hat{Y}}_{o}∼P_{G_{\theta }}}\left[\log\left(1-D_{\varphi }\left(c_{o},{\hat{Y}}_{o}\right)\right)\right]$$

(11) $$V\left(G_{\theta },D_{\omega }\right)=E_{\left(c_{\lambda },Y_{\lambda }\right)∼P_{\mathrm{phy}}}\left[\log D_{\omega }\left(c_{\lambda },Y_{\lambda }\right)\right]+E_{c_{\lambda }∼P_{\mathrm{phy}},{\hat{Y}}_{\lambda }∼P_{G_{\theta }}}\left[\log\left(1-D_{\omega }\left(c_{\lambda },{\hat{Y}}_{\lambda }\right)\right)\right]$$

The parameters for the generator and two discriminators are represented by θ, φ, and ω, which are updated in the training process. The comprehensive training procedure is outlined in Algorithm 1.


**Algorithm 1:** PICGAN-based car-following model training
1Assign initial values to the parameters θ for the generator ($G_{\theta }$), and to φ and ω for the discriminators ($D_{\varphi }$ and $D_{\omega }$);**In each training iteration**: 2Sample condition $c_{o}$ and output sequence $Y_{o}$ from observed training set;3Get the real samples $\left\{\left(c_{o}^{1},Y_{o}^{1}\right),\left(c_{o}^{2},Y_{o}^{2}\right),\ldots ,\left(c_{o}^{m},Y_{o}^{m}\right)\right\}$;4Sample input sequence $X_{o}$ and condition $c_{o}$ from observed training set, and generate output sequence ${\hat{Y}}_{o}$ by $G_{\theta }\left(X_{o}\right)$;5Get the generated samples $\left\{\left(c_{o}^{1},{\hat{Y}}_{o}^{1}\right),\left(c_{o}^{2},{\hat{Y}}_{o}^{2}\right),\ldots ,\left(c_{o}^{m},{\hat{Y}}_{o}^{m}\right)\right\}$;6Update parameters φ of discriminator $D_{\varphi }$ to increase $D_{\varphi }\left(c_{o},Y_{o}\right)$ and decrease $D_{\varphi }\left(c_{o},{\hat{Y}}_{o}\right)$,i.e., maximize $\hat{V}=\frac{1}{m}\sum_{i=1}^{m}\log D_{\varphi }\left(c_{o}^{i},Y_{o}^{i}\right)+\frac{1}{m}\sum_{i=1}^{m}\log\left(1-D_{\varphi }\left(c_{o}^{i},{\hat{Y}}_{o}^{i}\right)\right)$,$\varphi \leftarrow \varphi +\eta \nabla \hat{V}\left(\varphi \right)$;7Update generator parameters θ to minimize $\hat{V}=\frac{1}{m}\sum_{i=1}^{m}\log\left(1-D_{\varphi }\left(c_{o}^{i},G_{\theta }\left(X_{o}^{i}\right)\right)\right)$,$\theta \leftarrow \theta -\eta \nabla \hat{V}\left(\theta \right)$;8Sample condition $c_{\lambda }$ and output sequence $Y_{\lambda }$ from physics-based set;9Get the real samples $\left\{\left(c_{\lambda }^{1},Y_{\lambda }^{1}\right),\left(c_{\lambda }^{2},Y_{\lambda }^{2}\right),\ldots ,\left(c_{\lambda }^{m},Y_{\lambda }^{m}\right)\right\}$;10Sample input sequence $X_{\lambda }$ and condition $c_{\lambda }$ from physics-based set, and generate output sequence ${\hat{Y}}_{\lambda }$ by $G_{\theta }\left(X_{\lambda }\right)$;11Get the generated samples $\left\{\left(c_{\lambda }^{1},{\hat{Y}}_{\lambda }^{1}\right),\left(c_{\lambda }^{2},{\hat{Y}}_{\lambda }^{2}\right),\ldots ,\left(c_{\lambda }^{m},{\hat{Y}}_{\lambda }^{m}\right)\right\}$;12Update parameters ω of discriminator $D_{\omega }$ to increase $D_{\omega }\left(c_{\lambda },Y_{\lambda }\right)$ and decrease $D_{\omega }\left(c_{\lambda },{\hat{Y}}_{\lambda }\right)$,i.e., maximize $\hat{V}=\frac{1}{m}\sum_{i=1}^{m}\log D_{\omega }\left(c_{\lambda }^{i},Y_{\lambda }^{i}\right)+\frac{1}{m}\sum_{i=1}^{m}\log\left(1-D_{\omega }\left(c_{\lambda }^{i},{\hat{Y}}_{\lambda }^{i}\right)\right)$,$\omega \leftarrow \omega +\eta \nabla \hat{V}\left(\omega \right)$;13Update generator parameters θ to minimize $\hat{V}=\frac{1}{m}\sum_{i=1}^{m}\log\left(1-D_{\omega }\left(c_{\lambda }^{i},G_{\theta }\left(X_{\lambda }^{i}\right)\right)\right)$,

$\theta \leftarrow \theta -\eta \nabla \hat{V}\left(\theta \right)$





#### 3.3.2. Generator Structure within PICGAN

**Figure 3:**
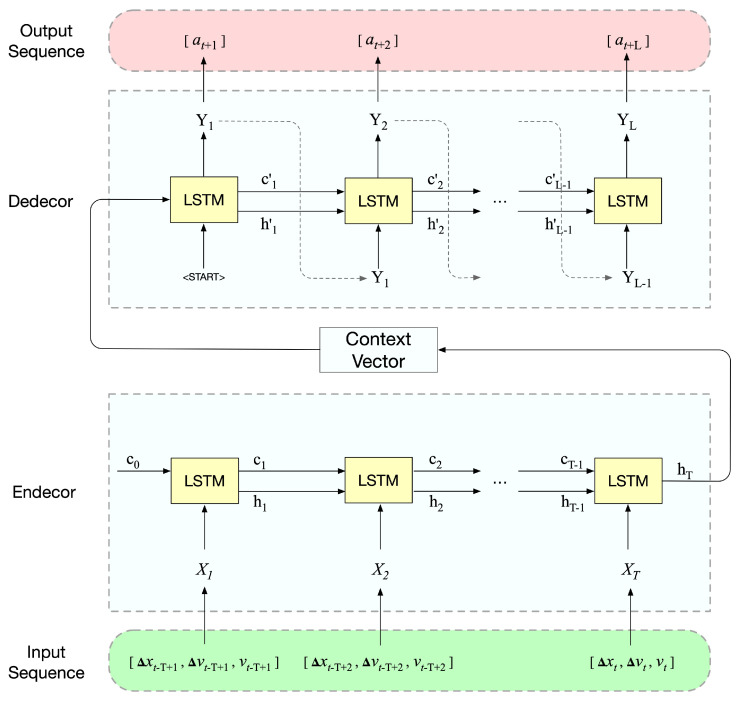
Generator structure.

The generator ($G_{\theta }$) in the proposed architecture utilizes an encoder–decoder structure, as illustrated in Figure 3. The structure of this generator corresponds with the sequence-to-sequence learning (Seq2Seq) car-following model presented in our earlier research [38]. This model takes multi-step driving states as input and generates multi-step actions as output. A comprehensive description of the fundamental framework of the Seq2Seq model and the long short-term memory (LSTM) unit can be found in that paper. In accordance with the decision-making process investigated in [38], we select distance- and speed-related factors for input and designated acceleration as output. As such, we assigned gap distance (Δx), relative speed (Δv), and speed (*v*) as input variables, while acceleration (*a*) acts as the output variable. Additionally, the generator’s input and output are sequences with lengths T and L, respectively. The mapping function is expressed in Equation (12):

(12) $$\begin{array}{cc} a_{t+1},\ldots ,a_{t+L}= & f(\Delta x_{t-T+1},\ldots ,\Delta x_{t},\\ \; & \;\Delta v_{t-T+1},\ldots ,\Delta v_{t},\\ \; & \;v_{t-T+1},\ldots ,v_{t}) \end{array}$$

#### 3.3.3. Discriminator Structure within PICGAN

**Figure 4:**
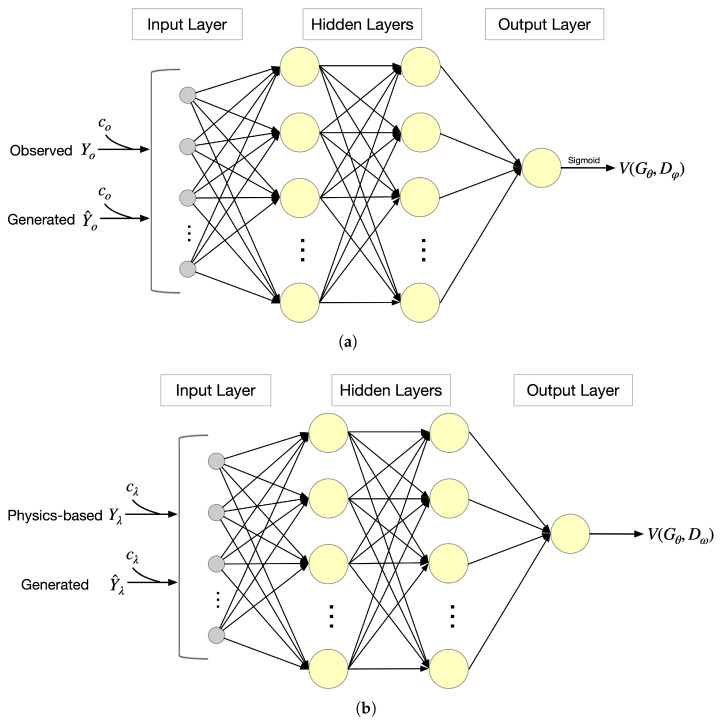
Discriminator structure: (**a**) Discriminator $D_{\varphi }$ evaluates output sequences based on observed samples. (**b**) Discriminator $D_{\omega }$ evaluates output sequences based on physics-based samples.

The proposed PICGAN architecture involves two discriminators, both of which employ a fully connected neural network structure, as shown in Figure 4, comprising two hidden layers. A sigmoid function is utilized in the output layer. The inputs fed into these discriminators consist of a condition and either real or generated actions relating to future time steps. The condition is determined by the observed actions or actions derived from a physics-based model from previous time steps. Each discriminator outputs a scalar value, representing a distinct probability. For $D_{\varphi }$, this probability signifies whether an action is real or generated, while for $D_{\omega }$, it indicates the likelihood of an action being sourced from the physics-based model or generated data.

## 4. Results

### 4.1. Data Preparation

The NGSIM dataset [57], known for its high-fidelity trajectory data, is widely used in traffic analysis and microsimulation research. In this study, we focused on the I-80 dataset, which was collected from eastbound US Interstate 80 in Emeryville, California. The dataset consists of six regular lanes and one high-occupancy vehicle (HOV) lane, spanning a 500-m area and three 15-min periods during congestion buildup and peak congestion on 15 June 2005. To ensure data consistency with vehicle kinematics and microscopic traffic dynamics, we employed the “traffic-informed” method proposed by Montanino and Punzo [58,59] for NGSIM data reconstruction. We excluded the HOV lane and focused on trajectory data from the regular lanes (Lanes 2–6) to avoid any atypical traffic behavior.

We extracted 1386 car-following events using the following criteria:

- Gap distance less than 120 m, to avoid free-flow traffic conditions.
- Vehicle length less than 5 m, to exclude trucks.
- Car-following duration of no less than 30 s continuously, to minimize the influence of lane changing.

**Figure 5:**
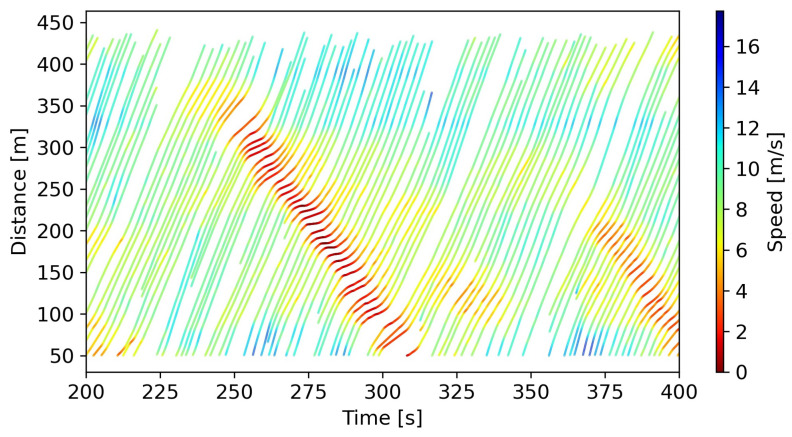
Space–time diagram (some trajectories from Lane 2).

With data preparation, we trained and tested our proposed hybrid car-following model and baseline models. To ensure the independence of the testing process, we used car-following events from Lane 2 (332 events) for testing and events from Lanes 3–6 (1054 events) for training. Figure 5 presents a portion of the trajectories in Lane 2.

### 4.2. Model Training

In the PICGAN, the physics-based model requires pre-training, which involves calibrating the parameters using the training dataset. The genetic algorithm (GA) [60] was employed in our research, as it is a popular calibration method with the ability to avoid local minima and achieve the global optimum through stochastic global search [61]. The detailed calibration procedure can be found in our previous study [62]. The calibrated values for the IDM’s parameters are presented in Table 1.

The training of the PICGAN car-following model was conducted using the process outlined in Algorithm 1 and executed in Python. Drawing from the earlier training of CGAN [45], we established the lengths of the input sequence and output sequence for the generator as 5 s and 1 s, respectively, that is, T=50 and L=10. Hyperparameters were tuned through experimental optimization as follows:

- Training Iterations: The learning algorithm iterates through the complete training dataset for a total of 5000 times (epochs), and each batch contains 128 instances. The performance of the generator was evaluated after each iteration to ensure the optimal parameters were captured.
- Neuron Configuration: Within the encoder–decoder architecture of the generator, there are 32 neurons in the LSTM units. In contrast, the hidden layers of the discriminators are configured with 64 neurons.
- Activation Function: The generator employs the hyperbolic tangent function, denoted as tanh(·), as its activation function. However, for the discriminators, the hidden layers utilize the leaky ReLU function, while the output layers use the sigmoid function.
- Optimizer Selection: The Adam optimizer [63], as it is efficient in training deep learning car-following models [38,44,45], was chosen. Its parameters were set as follows: learning rate (lr) = 0.00005, first moment estimate ($beta_{1}$) = 0.9, second moment estimate ($beta_{2}$) = 0.999, smoothing term (epsilon) = 1e-08, and decay = 0.0.

### 4.3. Model Performance

Based on the action produced, we applied the rule of discrete-time kinematics to deduce the predicted velocity and position, which are elaborated in Equation (13).

(13) $$\left\{\begin{array}{c} {\hat{v}}_{t+\Delta t}={\hat{v}}_{t}+{\hat{a}}_{t+\Delta t}\Delta t\\ {\hat{x}}_{t+\Delta t}={\hat{x}}_{t}+{\hat{v}}_{t}\Delta t+\frac{1}{2}{\hat{a}}_{t+\Delta t}\Delta t^{2} \end{array}\right.$$

where $\hat{a}$ refers to the generated action, specifically, acceleration. The predicted velocity and position are represented by $\hat{v}$ and $\hat{x}$, respectively. Meanwhile, *x* denotes the observed position.

The mean squared error (MSE) is a widely used measure for evaluating the accuracy of trajectory prediction tasks, making it a suitable metric for assessing the model’s performance. To gauge the accuracy of the trajectory prediction directly, we used space–time data to compute the MSE, as demonstrated in Equation (14).

(14) $$\mathrm{MSE}=\frac{1}{M}\sum_{t=1}^{M}{\left[x_{t}-{\hat{x}}_{t}\right]}^{2}$$

The IDM is a well-established car-following model often employed as a benchmark for comparing deep learning car-following models [36,38,39]. Thus, we utilize it as a baseline to assess the performance of the proposed model. Moreover, we contrast PICGAN with previously developed deep learning car-following models, including Seq2Seq [38] and CGAN [45].

**Figure 6:**
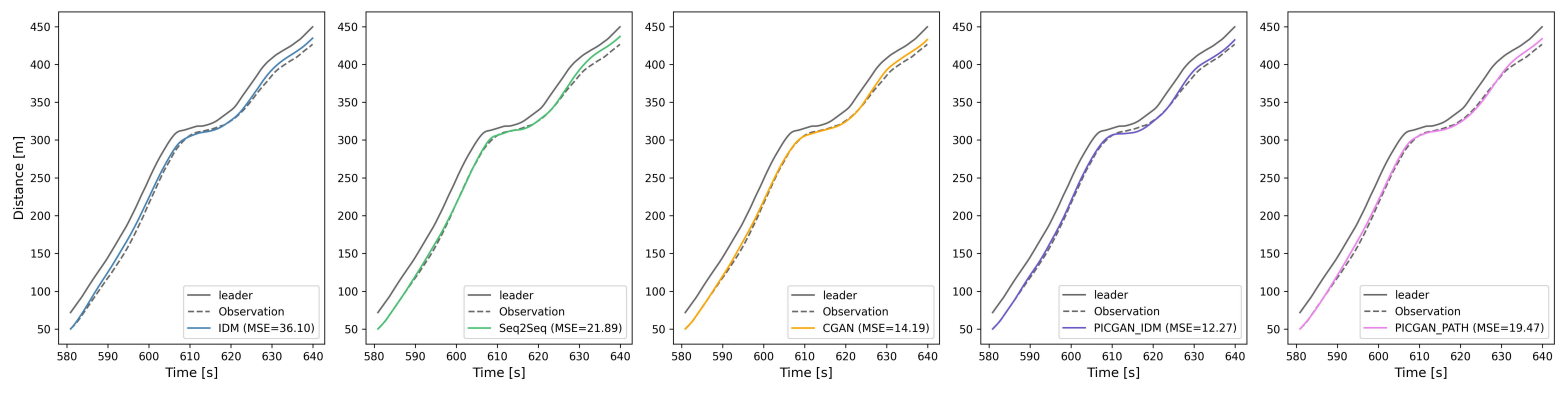
Space–time diagrams comparing observed and simulated trajectories of Vehicle 1898.

**Figure 7:**
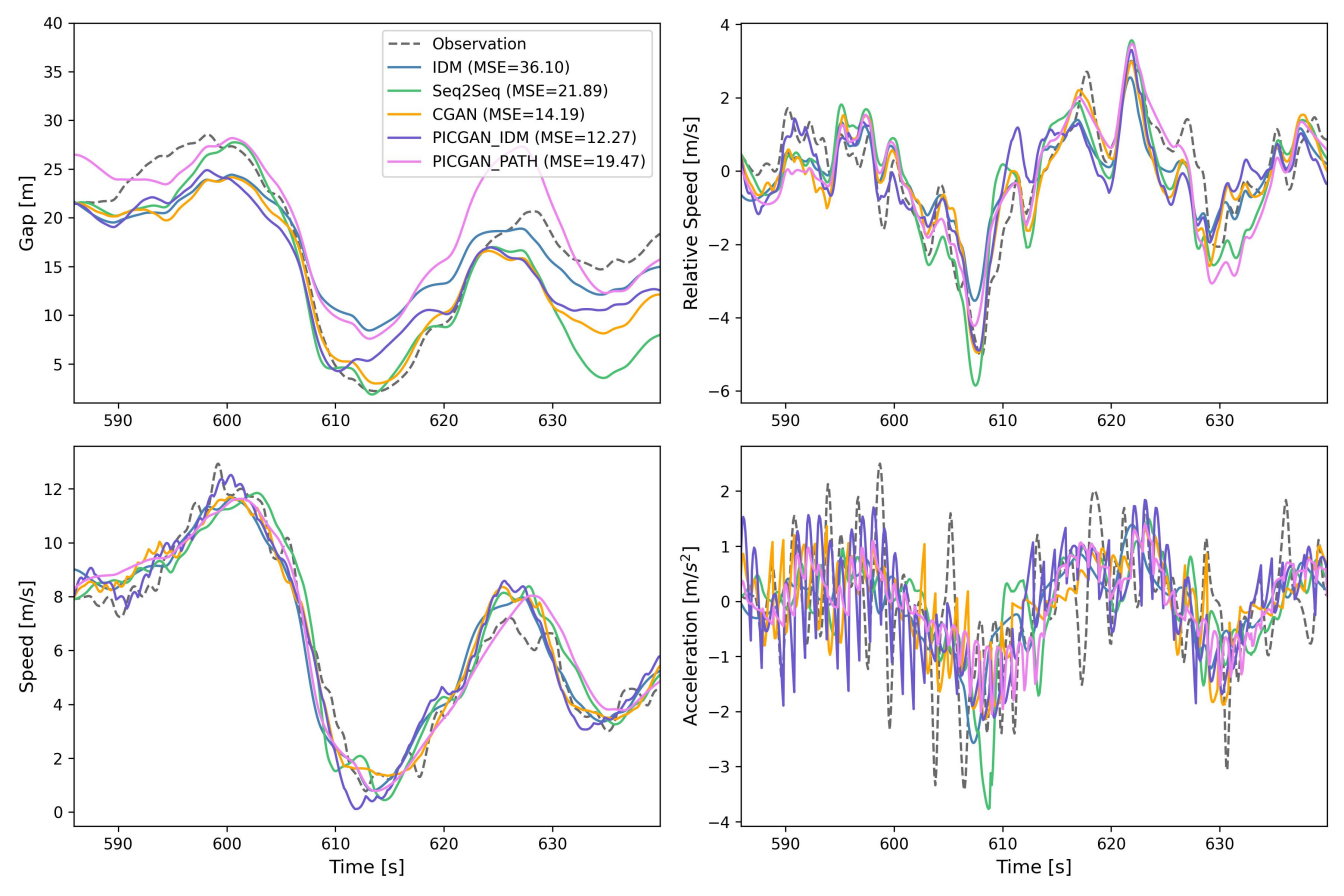
Profiles for Vehicle 1898 in terms of gap, relative speed, speed, and acceleration for models’ comparison.

In the PICGAN modeling, two physics-based models are utilized, respectively: IDM for modeling HVs, denoted as PICGAN_IDM, and CACC from the PATH laboratory (abbreviated as PATH) for modeling CAVs, denoted as PICGAN_PATH. To visually demonstrate the performance of these models, we randomly selected a car-following event (with Vehicle 1898 as the subject vehicle), and we depict the space–time diagrams in Figure 6. The reproduced trajectories using IDM, Seq2Seq, CGAN, PICGAN_IDM, and PICGAN_PATH were compared to the observed data, yielding MSE values of 36.10, 21.89, 14.19, 12.27, and 19.47, respectively. Figure 7 displays the trajectory profiles, highlighting the differences in gap distance, relative speed, speed, and acceleration among the various models. The results indicate that PICGAN_IDM achieved the highest accuracy, while PICGAN_PATH exhibited substantial performance, comparable to Seq2Seq.

To provide a comprehensive comparison, we performed a statistical analysis of the MSE values for the test dataset, which consists of 332 car-following events. The results are displayed in Table 2. The mean MSE values for IDM, Seq2Seq, CGAN, PICGAN_IDM, and PICGAN_PATH were 26.74, 21.60, 19.58, 18.47, and 22.69, respectively. Additionally, the standard deviation and percentiles offer further insights into the models’ performance.

Previous studies have demonstrated the efficacy of IDM in achieving reliable car-following performance [62], Seq2Seq in accurately simulating human driving behavior [38], and CGAN in generating a diverse range of car-following strategies [45]. The comparative analysis reveals that PICGAN_IDM surpasses these models, indicating the value of incorporating both observational and physics-based information. This finding aligns with the premise that a pre-trained IDM contributes to the hybrid model’s capacity to fit human driving behavior. Furthermore, while PICGAN_PATH is primarily intended for modeling CAVs, it demonstrates a moderate level of prediction accuracy for human driving behavior. Thus, the hybrid car-following model (PICGAN) offers superior action prediction accuracy, highlighting the benefits of combining physics-based and data-driven approaches.

### 4.4. Platoon Simulation

To delve deeper into the capabilities of the proposed PICGAN_PATH model, specifically tailored for CAVs, we carried out a platoon simulation. This strategy is critical for assessing the performance of car-following models. Unlike reproducing the trajectory of vehicle pairs, platoon simulation presents a unique challenge. In the process of reproducing trajectories, the anticipated action depends on the real-time states of the vehicle in front. But in the context of platoon simulation, all the subsequent vehicles, excluding the first one, are simulated based on their initial states and the simulation outcomes of their immediate predecessors. This means that any errors arising from the control model could possibly be compounded, providing a stringent test for the model’s effectiveness.

The configuration of our simulation takes inspiration from the study carried out by [64]. The platoon comprised 100 vehicles, and the first vehicle served as a leader under external control. The length of all vehicles was set to be 5 m. The simulation ran for a duration of 2000 s, updating every 0.1 s, which is reflective of the resolution of the field data. For the initial 50 s, the leading vehicle maintained a steady speed of v=15.3m/s, after which it slowed down to v=14.0m/s at a deceleration rate of $a=-0.65\;m/s^{2}$, maintaining this speed until the end of the simulation. The rest of the vehicles were considered to be in an equilibrium state with a speed of $v^{e}=15.3\;m/s$. Taking into account prior research [45,64], the corresponding equilibrium gap distance ($\Delta x^{e}$) was derived from the intelligent driver model (IDM). Given that both the relative speed and acceleration at equilibrium were zero, these values were inserted into Equations (1) and (2) to derive $\Delta x^{e}$, as represented in Equation (15). With the help of the calibrated parameters found in Table 1, the initial gap distance between all adjacent vehicles was determined to be 27.02 m.

(15) $$\Delta x^{e}=\frac{s_{0}+v^{e}t_{0}}{\sqrt{1-{\left(\frac{v^{e}}{\tilde{v}}\right)}^{4}}},$$

**Figure 8:**
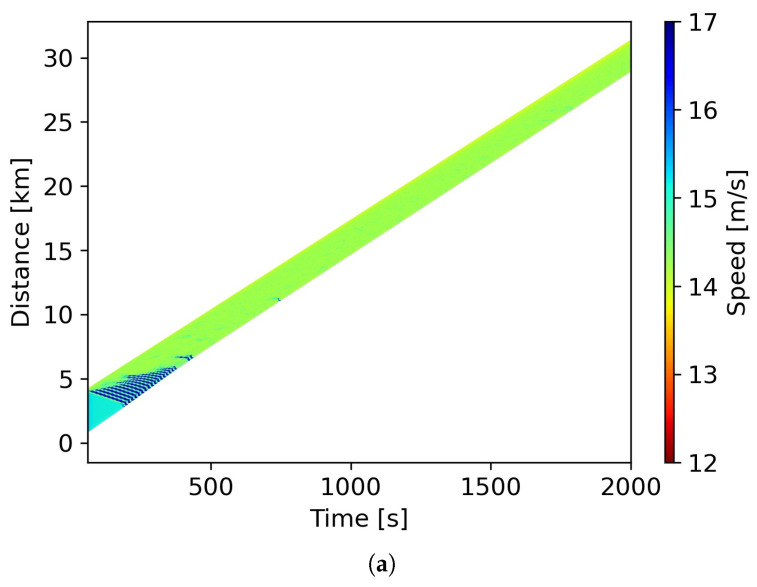

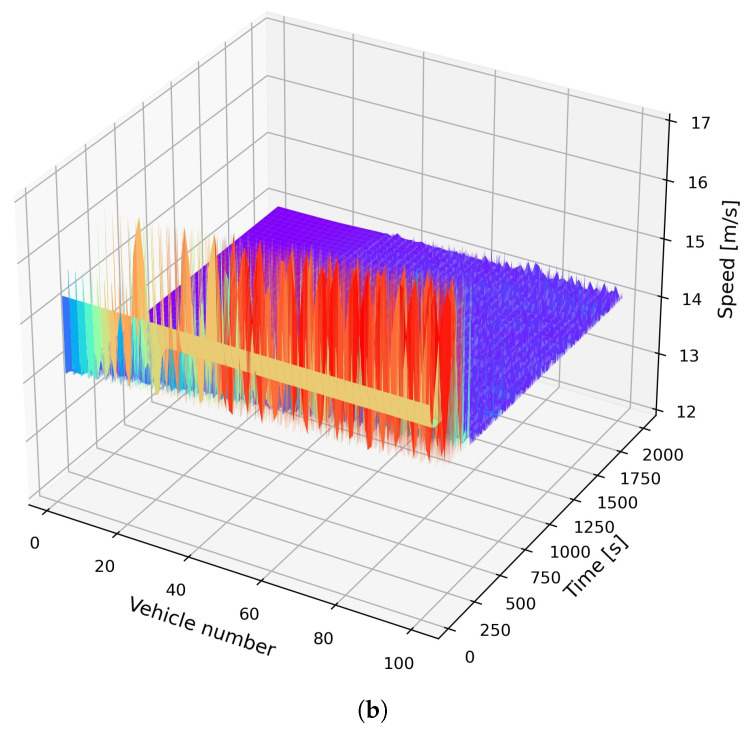
Results of the platoon simulation: (**a**) Diagram depicting the space–time relationship. (**b**) Evolution of speed over time.

Figure 8 presents the space–time and evolution diagrams. Both diagrams indicate that due to the deceleration of the first vehicle, the following vehicles in the platoon experienced traffic fluctuations for a short while. This is a typical event as the disturbance propagates towards the rear of the platoon. Shortly thereafter, the trajectories stabilized with diminished oscillation, leading to weaker speed and gap variances over time.

**Figure 9:**
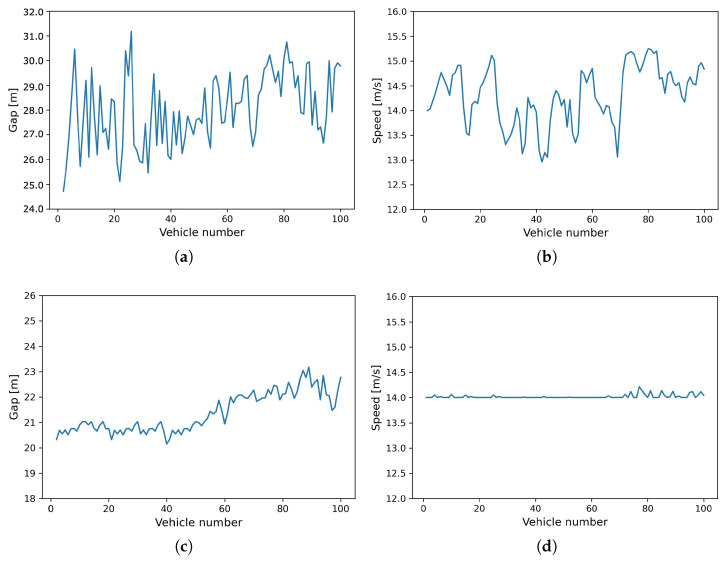
Comparison of snapshot profiles at 2000 s: (**a**) Snapshot of gap distance simulated by CGAN model. (**b**) Snapshot of speed simulated by CGAN model. (**c**) Snapshot of gap distance simulated by PICGAN_PATH model. (**d**) Snapshot of speed simulated by PICGAN_PATH model.

In order to further evaluate the stability of the proposed model specifically designed for CAVs, we scrutinized the final state of the platoon simulation. Figure 9 offers a comparative illustration of the simulated trajectories by both CGAN and PICGAN_PATH models at the 2000 s mark. This comparison reveals that vehicles simulated by both models do not have a uniform state. Instead, variations in their speed and gap distances are observed, but within specific ranges. Notably, the PICGAN_PATH model exhibits less pronounced variations than the CGAN model, indicating superior stability. This platoon simulation demonstrates the effectiveness of the proposed PICGAN_PATH model in the control of CAVs under realistic, continuous traffic flow.

### 4.5. Mixed Traffic Flow Simulation

Given that the PICGAN_IDM and PICGAN_PATH models were specifically developed for HVs and CAVs, respectively, it is crucial to evaluate their performance within a mixed traffic flow context. Mixed traffic flow simulation can be implemented using a periodic boundary condition, a method outlined by [65]. This method of experimentation is essential for evaluating the stability of traffic flow [66], and is in line with the presumption of an endless vehicle platoon in theoretical stability evaluations. This research utilizes the same periodic boundary condition to substantiate the effectiveness of the PICGAN model.

**Figure 10:**
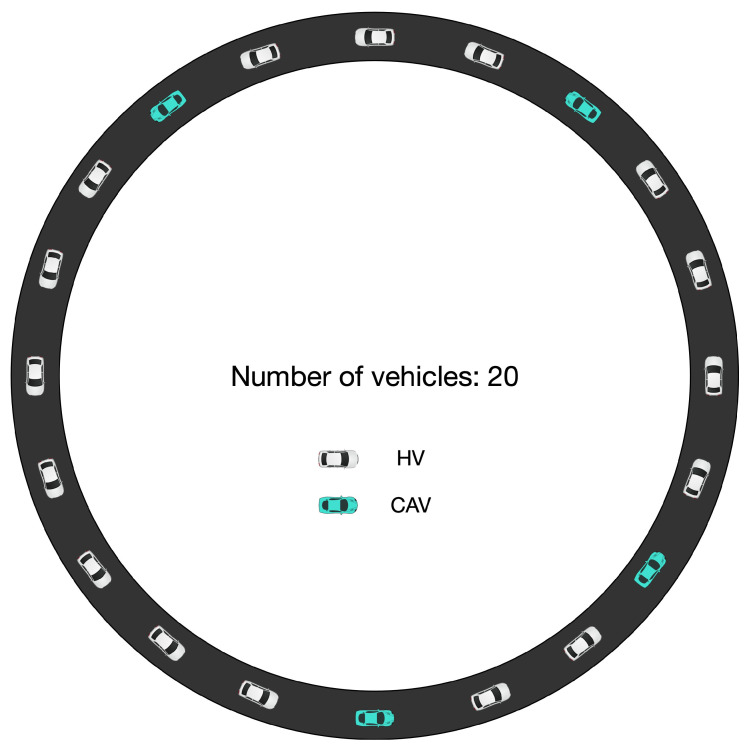
Diagram illustrating the periodic boundary condition in a scenario of mixed traffic flow, using a 20% CAVs penetration rate as an example.

We employed a circular road void of ramps, accommodating 20 vehicles operating in a head-to-tail formation. The vehicles were assumed to be 5 m long, mirroring the assumption in the platoon simulation, with initial speeds ($v^{e}$) and gap distances ($\Delta x^{e}$) of 15.3 m/s and 27.02 m. This configuration resulted in a circular road length of 640.4 m. At the point of 50 s into the simulation, a vehicle was chosen at random to experience a disruption, slowing down at a rate of 0.65 $m/s^{2}$ until it reached a speed of 14.0 m/s. Following this event, all vehicles continued to follow their respective car-following strategies for the remainder of the simulation. Both HVs and CAVs, which were randomly interspersed, were directed by the PICGAN_IDM and PICGAN_PATH models, respectively. The penetration rate for CAVs, represented by *p*, varied from 0% to 100% in increments of 20%, reflecting different configurations of mixed traffic flows. An illustration of this setup, with a 20% CAVs penetration rate as an example, is depicted in Figure 10.

**Figure 11:**
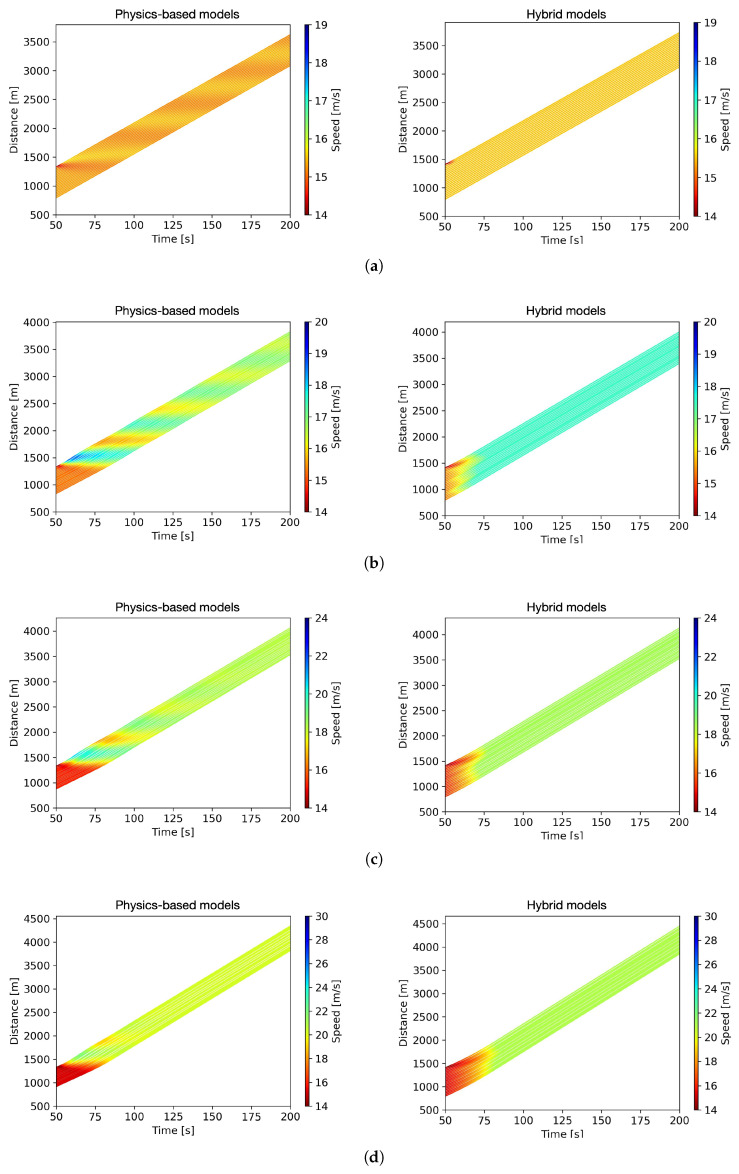

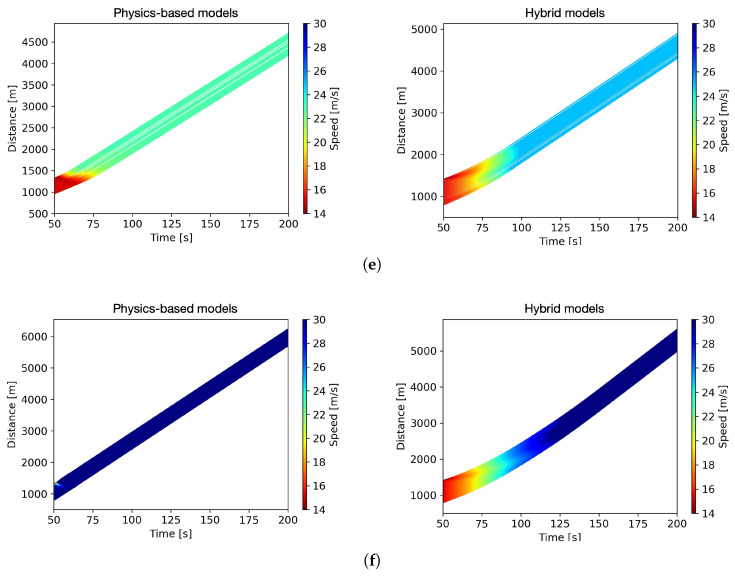
Space–time diagrams in terms of varying penetration rates of CAVs (*p*): (**a**) *p* = 0% (no CAVs). (**b**) *p* = 20% CAVs. (**c**) *p* = 40% CAVs. (**d**) *p* = 60% CAVs. (**e**) *p* = 80% CAVs. (**f**) *p* = 100% (all vehicles are CAVs).

The space–time diagrams simulated with physics-based models and hybrid models are compared in Figure 11. To streamline the presentation, the initial conditions spanning the first 50 s are not included for display. The simulation results indicate that trajectories modeled by the PICGAN framework exhibit only transient oscillations triggered by the initial perturbation, implying the stability of the PICGAN model. Moreover, as evident in Figure 11b–e, the introduction of CAVs into the traffic flow facilitates smoother trajectories. It can be inferred that the stability of mixed traffic flow can be enhanced with the PICGAN_PATH model.

A positive correlation is observed between the average vehicular speed and the penetration rate of CAVs. Compared to the outcomes produced by physics-based models, the simulation results of hybrid models indicate higher average operational speeds under various CAV penetration rates. This is visually evident from the color variations in the corresponding diagrams, implying that the hybrid models improve traffic efficiency.

**Figure 12:**
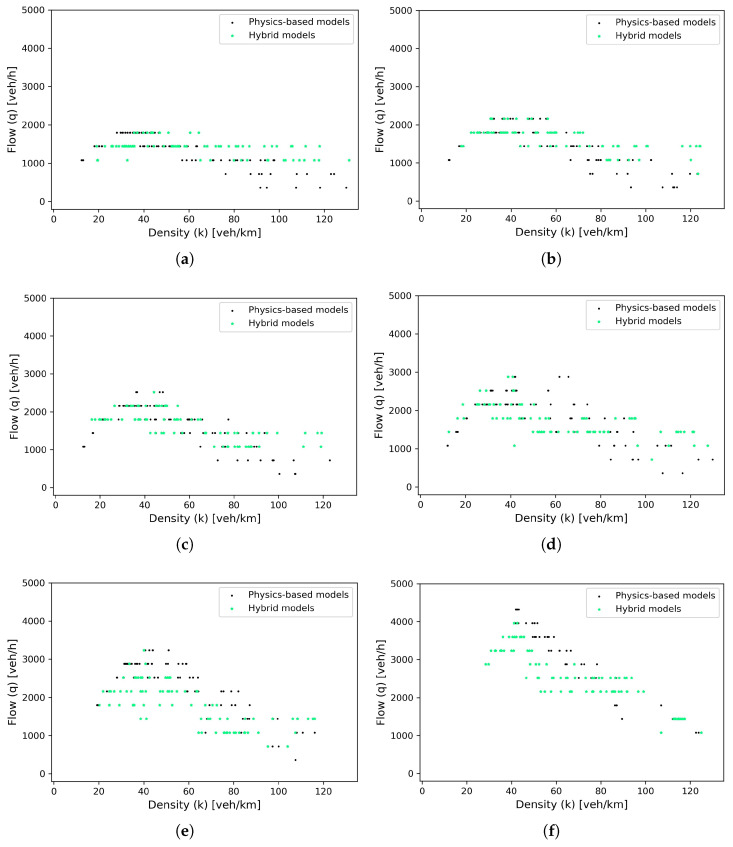
Fundamental diagrams in terms of varying penetration rates of CAVs (*p*): (**a**) *p* = 0% (no CAVs). (**b**) *p* = 20% CAVs. (**c**) *p* = 40% CAVs. (**d**) *p* = 60% CAVs. (**e**) *p* = 80% CAVs. (**f**) *p* = 100% (all vehicles are CAVs).

To evaluate the traffic capability, two virtual detectors were strategically placed at a distance of 100 m on the circular road. These detectors continuously monitor flow-density values at their respective positions throughout the traffic simulation. Consequently, the flow-density points can be visualized, as indicated by the green scatter points in the fundamental diagram in Figure 12. We subsequently compared the fundamental diagrams sourced from this simulation to those gathered from simulations that employed physics-based car-following models, which are represented by the black scatter points in the diagrams. The two sets of diagrams exhibit a high degree of consistency, thereby validating the suitability of the proposed hybrid model for traffic flow simulation. Furthermore, as the proportion of CAVs increases, a gradual rise in maximum capacity is observed in the fundamental diagrams. This trend further corroborates the effectiveness of the PICGAN_PATH model, as it signifies an improvement in the efficiency of the mixed traffic flow.

## 5. Conclusions

In this research, we have proposed a physics-informed conditional generative adversarial network (PICGAN), a novel hybrid model aiming to improve the performance of multi-step car-following modeling in mixed traffic scenarios. Our model effectively addresses the limitations of traditional physics-based and data-driven models. The PICGAN offers a more robust and adaptive solution by leveraging the inherent structure of GAN, which eliminates the need for an explicit weighting parameter typically needed to balance the contributions of different models.

The main findings of our research are as follows:

- The case study demonstrates that the PICGAN model exhibits superior performance in trajectory reproduction, effectively mimicking human driving behavior. Comparative analyses suggest that the PICGAN_IDM model holds promise as a strong contender to replace existing conventional models in trajectory prediction tasks.
- The PICGAN_PATH model successfully directed CAVs in platoon simulations, indicating its ability to manage consistent, continuous traffic flows with minimal errors. It also shows superior stability compared to the previously developed CGAN model, with less variation in vehicle speed and gap distances.
- The merits of the PICGAN model are further substantiated in a mixed traffic flow environment under periodic boundary conditions. The introduction of our hybrid model substantially improves the stability and efficiency of the mixed traffic flow.

In summary, the PICGAN framework, with its ability to deliver reliable and stable results, makes strides toward advancing efficient longitudinal control strategies for CAVs in real-world mixed traffic conditions. Future work could broaden the applicability of this hybrid model in the rapidly evolving autonomous vehicles landscape by exploring the potential of the PICGAN model in more complex traffic scenarios. Additionally, the model is primarily focused on longitudinal control, meaning it addresses car-following scenarios. As an area for further development, incorporating lateral control elements such as lane-changing behavior could be investigated. These are noteworthy considerations that will be addressed in our future research.

## Figures and Tables

**Table 1:** Calibrated parameters of the IDM.

	$\tilde{a}$	$\tilde{b}$	$\tilde{v}$	$t_{0}$	$s_{0}$
Calibrated value	2.02	1.43	22.89	1.40	2.75

**Table 2:** Statistics results of the performance.

Model	Mean (SD)	Min	Max	Percentile [25%, 50%, 75%]
IDM	26.74 (38.70)	1.21	374.28	[8.28, 14.87, 29.91]
Seq2Seq	21.60 (28.42)	0.73	233.29	[7.57, 12.27, 25.23]
CGAN	19.58 (22.73)	0.33	229.12	[6.66, 13.01, 24.18]
PICGAN_IDM	18.47 (18.08)	1.03	189.32	[6.54, 13.52, 25.90]
PICGAN_PATH	22.69 (23.99)	0.80	267.67	[7.17, 16.10, 29.57]

## Data Availability

Publicly available datasets were analyzed in this study. These data can be found at: https://data.transportation.gov/stories/s/i5zb-xe34; http://www.multitude-project.eu/exchange/101.html, (accessed on 15 May 2023).
